# Development of an analytical method for determination of lead and cadmium in biological materials by GFAAS using *Escherichia coli* as model substance

**DOI:** 10.1371/journal.pone.0267775

**Published:** 2022-05-03

**Authors:** Michelle Gende, Martina Schmeling

**Affiliations:** Department of Chemistry and Biochemistry, Loyola University Chicago, Chicago, IL, United States of America; Argonne National Laboratory, UNITED STATES

## Abstract

In this work, an analytical method was developed for the determination of lead and cadmium in biological samples using graphite furnace atomic absorption spectrometry. *Escherichia coli* (*E*. *coli*) was chosen as model substance for this purpose as it is readily available in most laboratories and can be quickly and easily prepared with a high turnaround rate. Four different sample preparation methods were initially evaluated with respect to percent recovery, limit of detection, and limit of quantification, and the most promising one was developed further. The final process involving microwave assisted digestion of the sample with nitric acid and hydrogen peroxide showed high recovery, repeatability, and specificity. The process was first applied to lead and then extended for the determination of cadmium in the same *E*. *coli* substrates. Finally, to validate the process, a certified references material was analyzed, and the results obtained were evaluated with respect to accuracy by comparing them to the reported ones.

## 1. Introduction

Metals play an important role in many biological processes ranging from the regulation of enzyme functions to neural signaling. (1) Only very few metals are toxic in any form to an organism with lead and cadmium being the most prominent ones [1–4].

Lead is among the earliest elements known to humankind and has been used extensively throughout history in many applications [5]. Pipes delivering water were often made of lead or a lead alloy, coins contained certain amounts of lead, and pigments made with lead salts were commonly used in paintings as well as cosmetics [5–8]. As a result of this ubiquitous use, the toxicity of lead has been investigated early on with the first observations dating as far back as the second century B.C. when the Greek physician Nikander noticed that colic and paralysis followed ingestion of lead [9]. Nevertheless, this did not deter people from using lead pots to boil grape juice to sweeten wine with the lead acetate present in this way. It was not until the end of the seventeenth century that the use of lead for the fortification of wine was banned in some regions of Europe as a result of its detrimental health effects [5,6,10]. The toxicity of lead stems from its divalent cation which can form strong bonds to sulfhydryl groups in proteins. This in turn can lead to distortion of enzymes, loss of protein structure, and development of endogenous opiate systems [11]. The toxic effects are most pronounced with the central nervous system as lead can mimic or compete with calcium. It has been documented that at even picomolar concentrations, lead causes interference with neuronal signaling by binding to cerebellar photokinase C instead of calcium [12]. This explains the paralysis the Greek physician first noticed back in the second century.

Today, the use of lead has been severely restricted, and people are exposed to lead mostly through tinted drinking water from old pipes or industrial mishandling, particularly of lead-acid batteries. To eliminate these exposures, regulations have been introduced to remove lead from paints, ceramic products, caulking, and pipe solder as well as battery recycling program. But even with these measures, lead poisoning is still common and according to the Institute for Health Metrics and Evaluation (IHME) it is estimated that in 2017, lead exposure accounted for 1.06 million deaths worldwide [13]. A major demographic that is affected disproportionately are children under the age of six due to the swallowing of dust and chips from deteriorating lead paint still present on many surfaces [14]. Blood concentrations of greater than 20 μg /dL of lead [15] are not uncommon and far exceeding the Food and Drug Administration’s (FDA) Interim Reference Levels (IRL) of 3 μg per day for children and 12.5 μg per day for adults, respectively [16].

In contrast to lead, the element cadmium was not known until the early 19^th^ century when Stromeyer and Hermann discovered it as an impurity of zinc oxide [17]. Cadmium sources are both natural through volcanic eruptions and erosion processes from rocks and soil and anthropogenic through industrial and agricultural uses mainly non-ferrous metal production, fossil fuel combustion, and phosphate fertilizers [18,19]. Cadmium is relatively mobile in the environment and bioconcentrates within the food chain. The main source of human exposure is via food predominantly through rice, leafy vegetables, and cereals [18,20,21]. Smokers have a higher exposure risk to the element as it bioconcentrates in tobacco [20]. With no known biological function and a very slow excretion rate, cadmium accumulates in organisms over time [2,20,22]. It gained notoriety through the Itai-Itai disease in Japan, where the local population was exposed to elevated cadmium levels in food, mainly rice, and water as a result of environmental release from an industrial plant [23]. Following this incidence, the toxicity of cadmium has been studied extensively over the last decades and it was found that cadmium affects mainly the kidneys and liver and has a high affinity towards metallothionine, a cadmium inducible protein that protects the cell by tightly binding to the cadmium ion [2,20,21,24]. Cadmium also interferes with the calcium metabolism therefore osteoporosis is not uncommon. Other impacts are on the pulmonary system, especially for people with high exposure rates [2,20,22].

Many countries, including the European Union, USA, Canada, and Australia introduced regulations limiting cadmium use to curb the exposure to this element. For instance, the US regulations specify a daily intake limit of 1x10^-3^μg/kg/day for food and 5x10^-4^μg/kg/day for water. The WHO recommends a threshold of 7μg/kg/body weight per week [18,25,26].

Several analytical methods are in use for the analysis of lead and cadmium in biological samples with atomic absorption spectrometry being one of the most common ones [27]. Specifically, graphite furnace absorption spectrometry (GFAAS) offers an attractive means to measure both elements as it not only has high sensitivity but also permits for analysis of small sample volumes between 5 and 20μl and has little interferences [27,28]. Other studies involving biological materials took advantage of these features and used GFAAS for the analysis of lead [29–31]. Nevertheless each of them was tailored for a specific material and no robust chemical measurement process currently exists, which can be adapted for a larger set of samples with comparable properties. Therefore, the goal of this study was to develop a robust chemical measurement process for lead and cadmium in biological samples which shows high sensitivity, accuracy, and precision. We selected *Escherichia coli* (*E*. *coli*) as a model substance for this purpose due to its ease of handling, ready availability, and its wide range of use in both the biological and life sciences. Several different sample preparation methods were tested and evaluated for their suitability. The methods tested included simple nitric acid dilution, addition of a matrix modifier to remove or suppress matrix effects, and acid digestion with or without microwave assistance. The final optimized process involves microwave assisted acid digestion and was initially developed for lead and then applied to cadmium. The performance of the procedure was then validated using certified reference material (BCR 679, white cabbage).

## 2. Materials and methods

### 2.1. Materials

All materials in this study were reagent grade or better. Nitric acid 70%, hydrogen peroxide 30 wt. %, lead, and cadmium nitrate were used to prepare digestion, and stock solutions were acquired from Sigma-Aldrich (St. Louis, MO, USA). A variety of salts used to determine the selectivity of the method were purchased either from Sigma-Aldrich (St. Louis, MO, USA), Thermo-Fisher (Waltham, MA, USA), or EMD (Chicago, IL, USA). Ultrapure water was obtained via a Purelab Flex purification system, >18 MΩ (ELGA® Labwater, High Wycombe, UK).

Digestions were carried out with polytetrafluoroethylene (PTFE) vessels (Anton Parr; Moline, IL, USA) and a Microwave oven (MW535OW; Samsung Corp, Suwon, Korea). Samples were analyzed by graphite furnace atomic absorption spectrometry (AA-7000, Shimadzu, Kyoto, Japan) at 283.3nm (Pb) and 228.8nm (Cd) wavelength, 0.7nm slit width, and 10mA lamp current. Samples analyzed on GFAA were pipetted in 20 μL increments into the atomization chamber and measured in triplicate following the furnace program shown in Table 2. (Shimadzu, Kyoto, Japan) Graphite pyrolytic coated tubes were purchased from Shimadzu and were exchanged after 500 firings to ensure high sensitivity. The hollow cathode tubes of 1.5” standard were purchased from Varsal. (Varsal LLC, Warminster, PA, USA).

All glassware was washed with a H_2_SO_4_ and HNO_3_ solution (1:3 ratio) by filling the glassware completely, soaking for an hour, and rinsing thoroughly with ultrapure water subsequently. The digestion vessels were cleaned twice between each digestion with 1 mL of 18 M HNO_3_, digested for one minute, and then rinsed excessively with ultrapure water.

### 2.2. Samples

The *E*. *coli* used in this study were grown from a BL21 cell line in Luria broth (LB) for 12–18 hours at 37°C. The cells were collected as pellets after centrifugation at 5,000 rpm for 5 minutes to remove the LB followed by two washes of 5 mL of 50 mM HEPES (4-(2-hydroxyethyl)-1-piperazineethanesulfonic acid, pH = 7.5; Sigma-Aldrich). After the second wash with HEPES, the *E*.*coli* cell pellets (ECP) were re-suspended in 500 μL of BSS (buffered saline solution, obtained from Loyola University Medical Center), to mimic the conditions used in biological settings. The ECP’s were then dispensed as 50 μL samples into different 1.5 mL centrifuge tubes, dried overnight at 95°C, and stored at room temperature until further use.

The certified reference material BCR 679 –White Cabbage was acquired from Sigma-Aldrich (St. Louis, MO, USA) and stored at room temperature until further use.

### 2.3. Instrumental parameter optimization

The furnace program needed to be adjusted to prevent the loss of lead and cadmium during the pyrolysis step. The final program is shown in Table 2 and was used for the determination of lead and cadmium in all samples described here. It was determined by comparing a sample containing no lead or cadmium (control) to a spiked sample containing 3.0 ng/mL Pb or Cd while varying the temperature of the pyrolysis stage ranging between 300°C and 800°C in 100°C increments until the recovery rate was close to 100% and reproducible.

### 2.4. Sample preparation procedures

**Fig 1:**
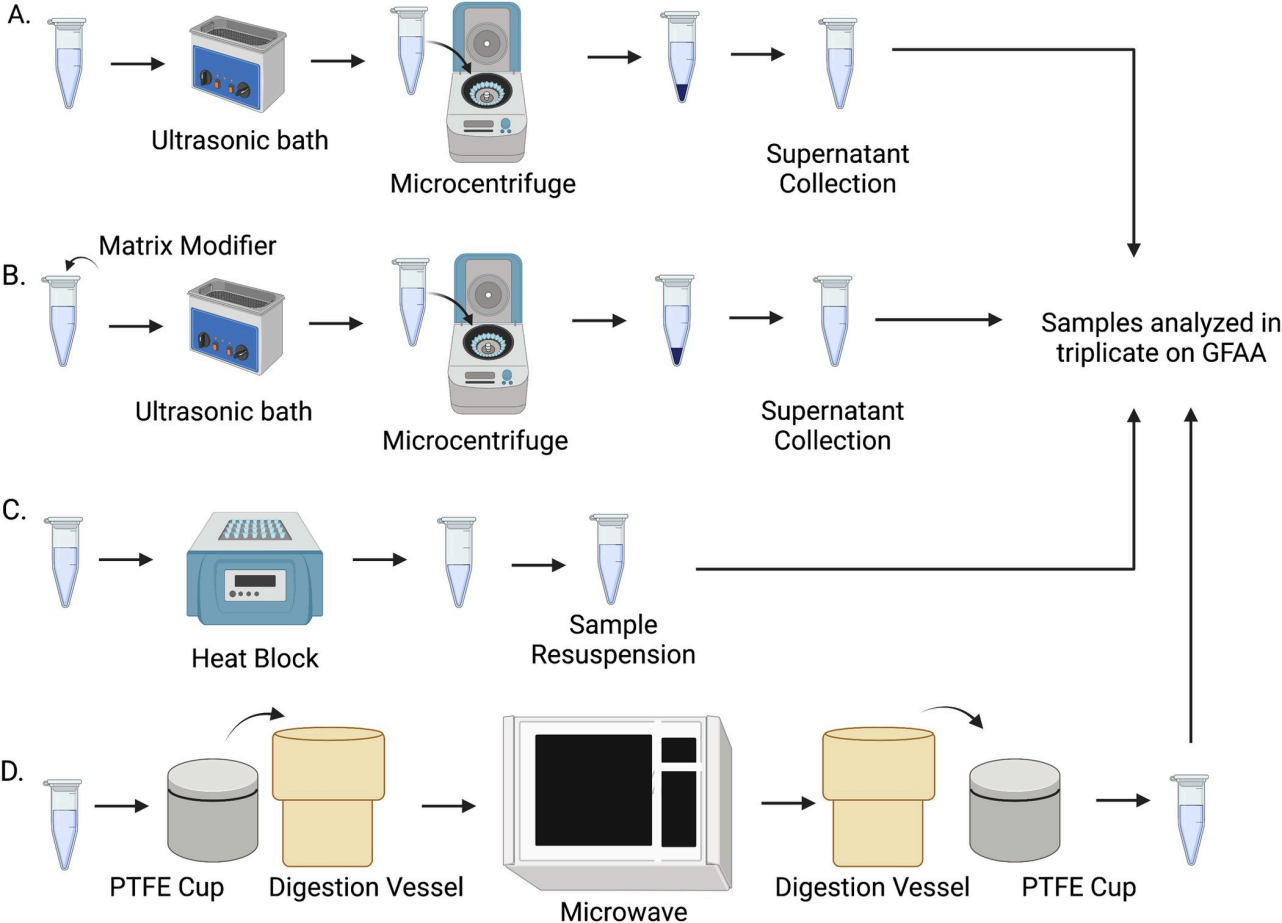
Flowchart of the four different sample preparation methodologies explored for the optimization of chemical process for the lead detection in biological materials A) Acidic dilution B) Addition of a matrix modifier C) Open-vessel acid digestion and D) Microwave assisted acid digestion.

For all methods, the ECPs were spiked with concentrations of 0, 3, 5, and 7 ng/mL of lead and cadmium, respectively, and diluted to a final volume of 1mL. These concentrations for lead and cadmium were chosen based on two criteria: 1) The concentrations should be at least five times higher than the detection limit of GFAA and 2) the concentration should cover the range reported for comparable biological matrics [32]. Reported lead concentrations ranged from 0.12 ± 0.02 μg/g in animal blood, 2.08 ± 0.03 μg/g in mussel tissue, and 13 ± 18 ng/g within the human lens. Cadmium concentrations were reported at 1.8 ± 0.1 μg/g in spinach leaves 0.353 ± 0.01 μg/g mussel tissue, & 20 ± 18 ng/g within the human lens [33–35]. The samples were treated in four different ways: 1) Addition of 500 μL of 10:1 HNO_3_ and H_2_O_2_ and 500 μL ultrapure water. Ultrasonication for 30 minutes, followed by centrifugation for five minutes at 5,000 rpm and collection of the supernatant for analysis. 2) Addition of 500 μL of 10:1 HNO_3_ and H_2_O_2_, plus 100 μL of 100 ng/mL of NH_4_H_2_PO_4_ and Mg(NO_3_)_2_ as matrix modifier, and 400 μL ultrapure water to obtain the final volume. The resulting solution was ultrasonicated for 30 minutes, followed by centrifugation for five minutes at 5,000 rpm and the supernatant was collected. 3) Addition of 500 μL of 10:1 HNO_3_ and H_2_O_2_ and 500 μL ultrapure water and digestion for 1 hour at 100°C using a digestion block and subsequent re-compensation to 1mL total volume with 50% v/v solution of 10:1 HNO_3_ to H_2_O_2_ and 4) Addition of 500 μL of 10:1 HNO_3_ to H_2_O_2_ and 500 μL ultrapure water and microwave assisted acid digestion in 23 mL PTFE sample cups at 600W power for 40 seconds. The four different sample preparation techniques are summarized in Fig 1.

### 2.5. Sample preparation procedure optimization

The microwave assisted acid digestion procedure was optimized further with respect to digestion time, power settings, and nitric acid to hydrogen peroxide ratio. This was done consecutively starting with the digestion time. For this, the samples with predetermined lead concentrations as described in section 2.4. were microwaved for 30, 40, 50, 60, 70, 80, and 90 seconds, respectively, and the percent recoveries were calculated.

Next, the microwave power setting was optimized. The spiked samples were digested starting with the lowest setting of 600W, the power increased in 120W increments until maximum power of 1200W, and the percent recoveries were calculated.

In the last step, the ratio between hydrogen peroxide and nitric acid in the digestion solution was optimized. The ratios 10:1, 6:1, 5:1, 4:1, and 2:1 HNO_3_ to H_2_O_2_ were tested using the spiked samples and for each the recoveries were calculated.

To determine the repeatability of the optimized method 12 ECP samples spiked with 4ng/ml of lead or cadmium and 3 controls containing only BSS were prepared using both the initial and optimized sample preparation process. The recoveries were calculated after subtractions of the blanks (controls).

The specificity of the optimized method was tested by adding known amounts of elements commonly found in biological materials. The elements and their concentrations are listed in Table 1. The elements were added first as single elements and then as a mixture containing all of them. The ECPs were then treated similarly than before, and the recoveries were calculated.

**Table 1:** Concentrations of metals used for the selectivity study.

Element	Amount Spiked
Na	380 ng/mL
Mg	380 ng/mL
K	380 ng/mL
Ca	380 ng/mL
Mn	60 ng/mL
Fe	120 ng/mL
Ni	75 ng/mL
Cu	120 ng/mL
Zn	60 ng/mL

### 2.6. Analysis of certified reference material

Lead and cadmium were analyzed in certified reference material. For this, a 0.1000g amount of the certified material BCR 679 –White Cabbage was weighed, and the optimized chemical measurement process followed. This was done for 15 subsamples for cadmium as required in the analysis certificate and 10 times for lead, which was not certified. In addition, 2 controls for each element were also measured.

## 3. Results and discussion

### 3.1. Instrumental parameter optimization

**Fig 2:**
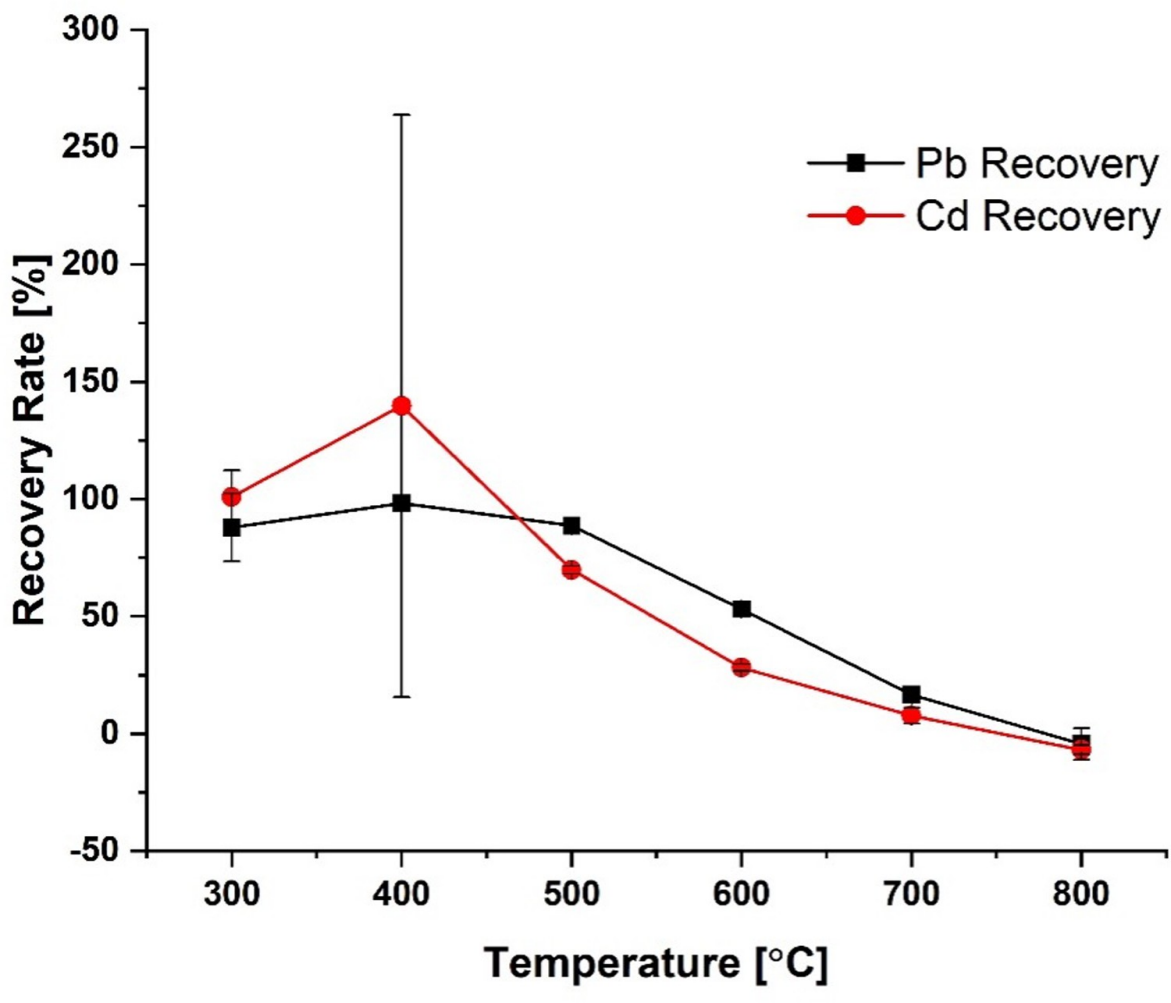
Optimization of the graphite furnace atomic absorption pyrolysis stage.

The graphite furnace program is important to ensure that the entire sample is atomized, and quantitative analysis occurs. The furnace program can be described as four different stages: the drying, pyrolysis, atomization, and the cleaning stage. Each of these plays a crucial role in the detection of the analyte. The first step or drying stage removes water from the sample; the pyrolysis stage combusts organic material; the next stage atomizes the analyte, and the last stage reconditions the furnace by removing any residual sample at high temperatures. Since this study involved a new chemical measurement process, it was necessary to optimize the furnace program. For this, several different settings were tested, and the percent recoveries were calculated. During the optimization of the furnace program, the pyrolysis stage was identified as the critical phase where a large amount of analyte was being lost, hence major focus was placed on the optimization of this stage. By lowering the temperature for this stage from 800°C to 400°C for lead and to 300⁰C for cadmium, the recovery rates improved to close to 100% for both elements. Fig 2 summarizes the optimization of the pyrolysis stage showing how the percent recovery for both elements is changing with temperature. Using a too high pyrolysis temperature most likely causes interaction between the element and the organic matrix of the sample reducing the element’s ability to become pyrolized^3^.

The furnace program having the best recovery rates for both elements is shown in Table 2. It comprises eight steps and reaches a maximum temperature of 2500° C.

**Table 2:** Optimal graphite furnace temperature program for the detection of lead and cadmium in *E*. *coli* used for microwave assisted acid digestion (optimal method).

Step		Time (sec)	Lead	Cadmium
			Temperature (C°)	Temperature (C°)
1	Drying	3	60	60
2		20	150	150
3		10	250	250
4	Pyrolysis	10	400	300
5		10	400	300
6		3	400	300
7	Atomization	3	2000	2250
8	Cleaning	2	2500	2500

### 3.2. Sample preparation method optimization

Four different sample preparation methods were evaluated for the determination of lead in biological samples using *E*. *coli* as model substance. The highest performing method was then optimized and subsequently applied to the determination of cadmium. The performance of each of these methods is summarized in Table 3 with respect to percent recovery, limit of detection, and limit of quantification. The methods evaluated were acid dilution, acid dilution with matrix modification, heat assisted open vessel acid digestion, and microwave assisted acid digestion.

**Table 3:** Performance of the four sample preparation methods for lead detection in *E*. *Coli*.

Method	Percent Recovery (%)	LOD (ng/mL)	LOQ (ng/mL)
Acidic dilution	59.2 ± 46.7	1.30 ± 0.124	4.34 ± 0.394
Acid dilution and matrix modifier	77.1 ± 35.3	1.04 ± 0.320	3.45 ± 1.06
Heat assisted open vessel digestion	162 ± 151	1.03 ± 0.236	3.44 ± 0.789
Microwave assisted acid digestion	74.4 ± 1.33	1.43 ± 0.232	4.75 ± 0.737

Dilution of the samples with nitric acid and hydrogen peroxide (10:1) was explored because it is simple, fast, and inexpensive. Not surprisingly, however, the *E*. *coli* samples had a low percent recovery with a large standard deviation and was not considered further.

The addition of a matrix modifier is often used to eliminate unwanted matrix mostly consisting of organic material without the loss of analyte [36]. In this case a combination of ammonium dihydrogen phosphate and magnesium nitrate was chosen as the matrix modifiers since this had success in determining both lead and cadmium in animal tissue, milk, eggs, and feedstuff [37,38]. In our application, the addition of a matrix modifiers performed had a decent percent recovery however it had a large standard deviation and was not considered a viable option for sample preparation. The next procedure investigated was heat assisted open vessel acid digestion, which is sometimes applied to more complex materials where dilution with acid and hydrogen peroxide is not sufficient to digest the analyte of interest. In case of the *E*. *coli* cell pellets, heat assisted open digestion showed a recovery rate well above 100% indicating contamination. This is in line with other experiments, which found open digestion to be not reliable for biological samples due to the high risk of contamination or losses as well as substantial standard deviations [39]. Hence this method was also rejected. The final approach involved microwave assisted acid digestion. This process has been reported to be performing well for biological samples as it allowed for complete digestion with only small amounts of reagents, minimum risk of contamination, and also showed high reproducibility [39,40]. For the *E*. *coli* samples investigated in this study, microwave assisted acid digestion provided the best initial performance and was therefore optimized further.

### 3.3. Optimization of microwave assisted acid digestion

In the previous section, it was found that microwave assisted acid digestion yielded the best results for the determination of lead in *E*. *coli* cell pellets. The method was optimized further by assessing digestion time, power applied, and the ratio of nitric acid to hydrogen peroxide.

**Fig 3:**
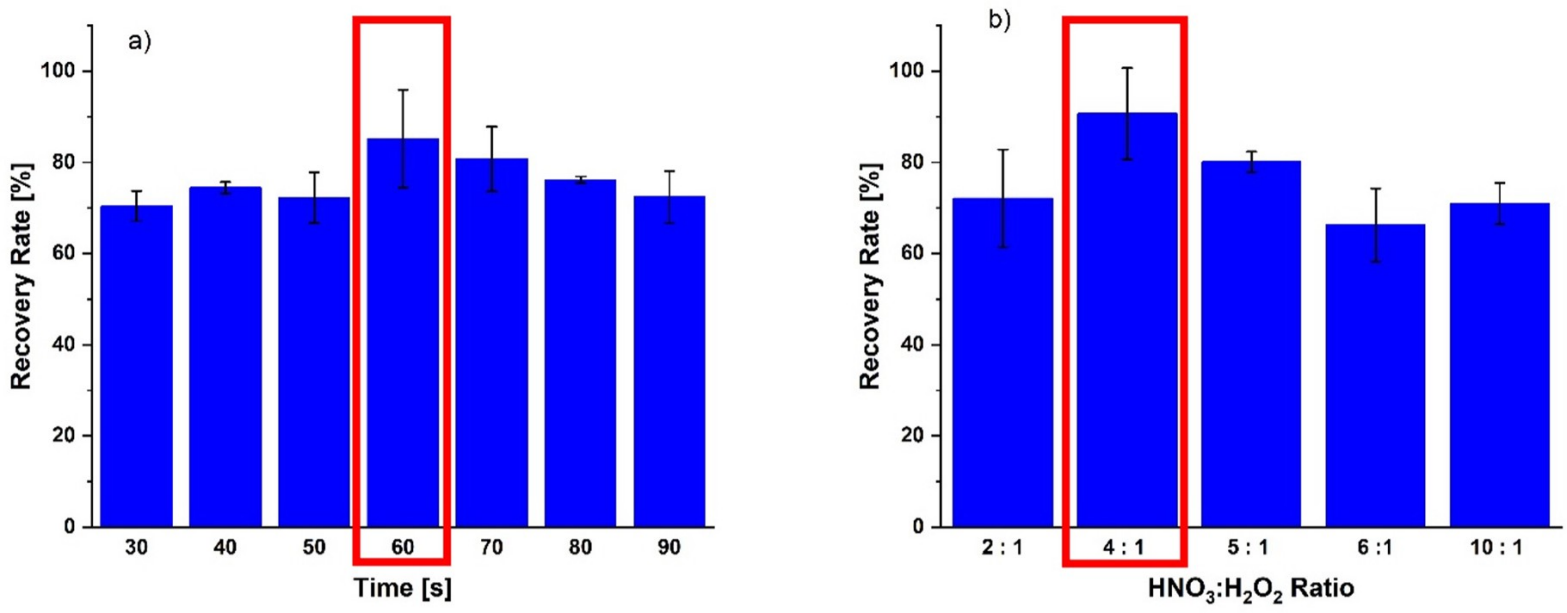
Optimization of microwave assisted acid digestion. a) time of digestion; b) hydrogen peroxide to nitric acid ratio. The optimal settings are boxed.

Eight different digestion times were tested ranging from 30–90 seconds and the percent recovery calculated for each of them. Fig 3A summarizes the applied power settings indicating that the highest recovery rate was obtained for a 60 second digestion time and this time was used for all subsequent experiments.

The next parameter investigated was the power setting of the microwave. Six different power settings were applied and as before, the percent recovery was used to assess the optimal one. No significant difference was observed between the different power settings, and it was decided to use the maximum power setting of 1200W going forward.

Finally, the ratio between hydrogen peroxide and nitric acid was assessed. As before the percent recovery was applied as a determinant of the highest performance and it was found that a 4:1 ratio between nitric acid and hydrogen peroxide yielded the best percent recovery with higher than 95%. Fig 3 displays the microwave times (3a, left) and nitric acid to hydrogen peroxide ratios (3b, right) tested highlighting the optimized settings by a box, and Table 4 contrasts the optimized microwave assisted acid digestion procedure for the determination of lead in *E*. *coli* with the initial one.

**Table 4:** Comparison between the optimized and initial microwave assisted acid digestion procedure with respect to recovery rates, limits of detection (LOD), limits of quantification (LOQ), and repeatability expressed as relative standard deviation between measurements.

Method	Percent Recovery (%)	LOD (ng/mL)	LOQ (ng/mL)	Repeatability [%]
Microwave assisted acid digestion: initial	7’6.7 ± 4.92	1.43 ± 0.233	4.753 ± 0.743	37.6%
Microwave assisted acid digestion: optimized	99.0 ± 9.46	0.393 ± 0.0462	1.31 ± 0.154	18.4%

The repeatability of the optimized process expressed as relative standard deviation between the measurements was determined by analyzing 12 samples containing the same amount of lead and 3 controls having no lead present. The repeatability of the optimized method was 18.4%, which is substantially better than the 37.6% repeatability obtained for the initial method. The somewhat higher relative standard deviation can most likely be attributed to the variable blank value of lead measured in the *E*. *coli* samples alone. The samples were stored in buffered saline solution (BSS) solution to simulate the chemical environment of biological samples and an average blank value of 1.27ng/mL ± 14.0% was found.

**Fig 4:**
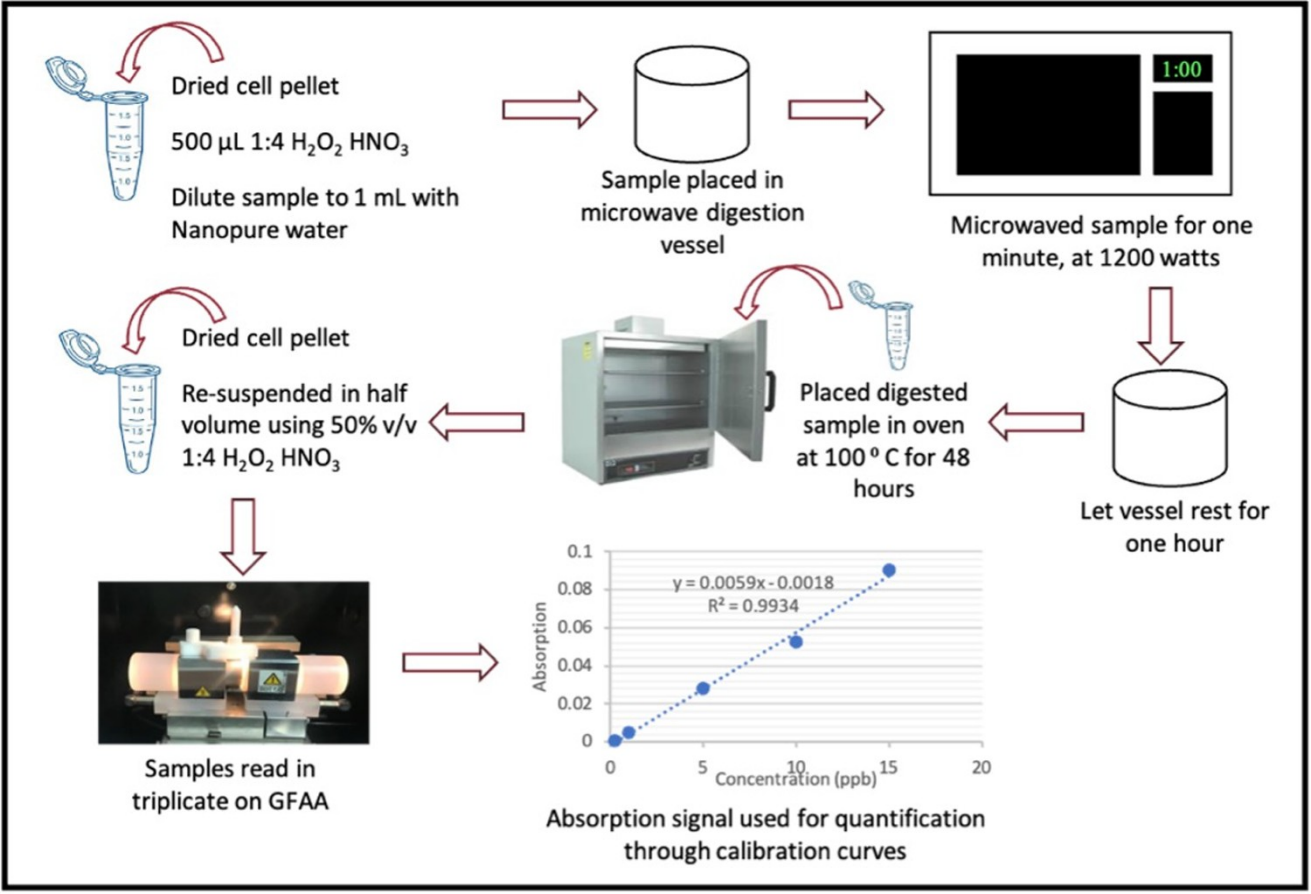
Optimized microwave assisted acid digestion for determination of lead in *E*. *coli*.

The finalized chemical measurement process is depicted in Fig 4 and can be summarized as follows: suspension of the biological sample (*E*. *coli*) in 500μL 4:1 HNO_3_:H_2_O_2_ and 500μL ultrapure water. Microwave digestion of this solution at 1200W for one minute. Transfer of the sample into a pre-cleaned and heat prove container and dry at 100°C for 48 hours. The drying and subsequent resuspension of the sample in half the volume pre-concentrates the sample in cases when elements of interest are close to or below limit of detection such as for *E*. *coli*. Final resuspension of the sample in 500 μL of 50% v/v 4:1 HNO_3_:H_2_O_2_ solution and subsequent analysis by GFAAS.

### 3.4. Application of the sample preparation method for cadmium and specificity of the method

Besides lead, cadmium is detrimental to organisms thus it was important to be able to determine cadmium with the same sensitivity in biological samples as lead. To do so the now optimized sample preparation method was applied for the measurement of cadmium in ECPs and the same parameter was determined for lead. Table 5 summarizes the results obtained and as can be seen, the method performed equally well for the determination of cadmium showing a high recovery rate of the element as well as low detection limits, quantification limits, and good repeatability.

**Table 5:** Performance of the developed and optimized sample preparation method for cadmium.

Element	Percent Recovery [%]	LOD [ng/mL]	LOQ [ng/ml]	Repeatability [%]
Cadmium	110 ± 11.6	0.646 ± 0.868	2.14 ± 0.283	10.9%

**Fig 5:**
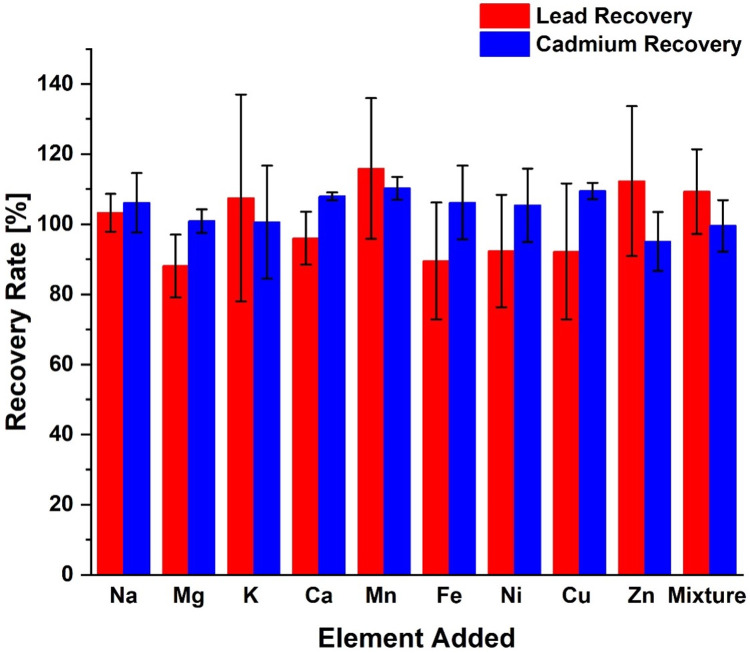
Average recovery rates for lead and cadmium in presence of potentially interfering elements. The concentration of those elements is shown in Table 1. Each element was investigated first individually and then a mixture of all elements combined.

Biological samples often contain large amounts of metabolic elements like sodium, potassium, chlorine, and iron, which potentially can interfere with the analysis of the desired analyte. Hence, it was important to determine how specific the developed chemical measurement process is with respect to determine elements of interest, in this case, lead and cadmium. A previous study was used to identify the potentially interfering elements and their concentrations are listed in Table 1 [41]. To evaluate the specificity of the method, the listed amounts of the potentially interfering elements were first added individually and then as a mixture. The recovery rates of lead and cadmium were measured. Fig 5 shows the results obtained in the form of recovery rates. It can be concluded that the developed chemical measurement process is robust and was not influenced by other elements present in the sample.

### 3.5. Analysis of certified reference material

The developed sample preparation method was validated by analyzing certified reference material. Unfortunately, biological certified reference materials reporting both cadmium and lead are not available, thus it was decided to select BCR 679 –white cabbage, which reported cadmium as one of the elements certified and matched closely enough the chemical consistency and composition of *E*. *coli* as well as other biological materials. Table 6 displays the results obtained with the required 15 subsamples from BCR 679 being analyzed for cadmium and an additional 10 subsamples analyzed for lead. As can be seen from the table, the cadmium amount determined is in good agreement with the certified one, thus showing a high accuracy of the developed chemical measurement process.

**Table 6:** Cadmium and lead concentrations measured in BCR 679 following the developed sample preparation method. Please note that only cadmium was certified.

Element	Mass Fraction of Developed Method (mg/kg)	Mass Fraction reported (mg/kg)	Number of Samples
Cadmium	1.59 ± 0.161	1.66 ± 0.699	15
Lead	0.204 ± 0.0393	Not reported	10

## 4. Conclusion

An analytical method for determination of lead and cadmium in biological materials was developed using *E*. *coli* as model substance. The method involved the exploration of four different sample preparation techniques, its subsequent optimization for lead detection and finally its application for cadmium determination. The final method consisted of microwave assisted digestion with a 4:1 mixture of HNO_3_:H_2_O_2_, 60 seconds digestion time at the highest power setting of 1200W, and subsequent analysis by GFAAS using a specifically tailored furnace program for lead and a separate one for cadmium. It had a high recovery rate and good repeatability for both elements and was not impacted by the presence of other elements in the sample. In a final step, the analytical method was validated by analyzing cadmium in certified reference material and proved to be suitable for this material as well.

## References

[1] Mehri A. Trace Elements in Human Nutrition (II)—An Update. International Journal of Preventive Medicine. 2020. doi: 10.4103/ijpvm.IJPVM_48_19 32042399 PMC6993532

[2] Genchi G, Sinicropi MS, Lauria G, Carocci A, Catalano A. The Effects of Cadmium Toxicity. International Journal of Environmental Research and Public Health. 2020;17(3782). doi: 10.3390/ijerph17113782 32466586 PMC7312803

[3] Flora G, Gupta D, Tiwari A. Toxicity of lead: A review with recent updates. Interdisciplinary Toxicology. 2012;5(2):47–58. doi: 10.2478/v10102-012-0009-2 23118587 PMC3485653

[4] bhat SA, Hassan T, Majod S. Heavy metal toxicity and their harmful effects on living organisms-review. International Journal of Medical Science and Diagnosis Research (UMSDR). 2019;3(1):2581–3935.

[5] Lessler MA. Lead and Lead poisoning from Antiquity to Modern Times. Ohio Journal of Science. 1988;88(3):78–84.

[6] Eisinger J. Lead and wine. Eberhard Gockel and the colica Pictonum. Medical History. 1982;26(3):279–302. doi: 10.1017/s0025727300041508 6750289 PMC1139187

[7] Cases JS, Sordo J. Lead: Chemistry, Analytical Aspects, Environmental Impact and Health Effects. Amsterdam, Boston, Heidelberg, London, New York, Oxford, Paris, San Diego, San Francisco, Singapore, Sydney and Tokyo.: Elsevier; 2006.

[8] Kamenov GD, Gulson BL. The Pb isotopic record of historical to modern human lead exposure. Science of the Total Environment. 2014;490:861–70. doi: 10.1016/j.scitotenv.2014.05.085 24907620

[9] Needleman H. Lead Poisoning. Annul Rev Med. 2004;55:209–22. doi: 10.1146/annurev.med.55.091902.103653 14746518

[10] Rhodes J. Sugar of Lead: A Deadly Sweetener Smithsonian Magazine 2012 [https://www.smithsonianmag.com/arts-culture/sugar-of-lead-a-deadly-sweetener-89984487/].

[11] Bailey C, Kichen I. Ontogenesis of proenkephalin products in rat striatum and the inhibitory effects of low-level lead exposure. Dev Brain Res. 1985;22:75–9. doi: 10.1016/0165-3806(85)90070-7 4041920

[12] Brown RK, Hingerty BE, Dewan JC. Pb(II)-catalysed cleavage of the sugar-phosphate backbone of yeast tRNA(Phe)-implications for lead toxicity and self-splicing RNA. Nature. 1983;303:543–6. doi: 10.1038/303543a0 6343887

[13] Lead poisoning and health. https://www.who.int/news-room/fact-sheets/detail/lead-poisoning-and-health.

[14] Committee on Measuring Lead in Critical Populations NRC. Measuring Lead Exposure in Infants, Children and other sensitive populations.: National Academic Press; 1993. 356 p. 25144057

[15] Tchounwou PB, Yedjou CG, Patolla AK, Sutton DJ. Heavy metals toxicity and the environment. EXS. 2012;101:133–64. doi: 10.1007/978-3-7643-8340-4_6 22945569 PMC4144270

[16] Administration FD. Lead in Food, Foodwares and Dietary Supplements https://www.fda.gov/food/metals-and-your-food/lead-food-foodwares-and-dietary-supplements#:~:text=Lead%20occurs%20in%20foods%20because,in%20food%2C%20including%20dietary%20supplements.2020.

[17] Britannica. The Editors of Encyclopaedia "cadmium": Encyclopedia Britannica; 2021 [https://www.britannica.com/science/cadmium].

[18] Review NCM-C. Background paper to meeting UNEP Governing Council 2003. file:///Users/michellegende/Desktop/Toxicity%20of%20lead-%20A%20review%20with%20recent%20updates.pdf2003.

[19] Organization WH. IPCS (International Programme on Chemical Safety) Environmental Health Criteria 134. Geneva, Switzerland1992.

[20] Bernard A. Cadmium and its adverse effects on human health. Indian J Med Res. 2008;128(4):557–64. 19106447

[21] Satarug S. Dietary Cadmium Intake and its Effects on Kidneys. Toxics. 2018;6(15). doi: 10.3390/toxics6010015 29534455 PMC5874788

[22] Rani A, Kumar A, Lal A, Pant M. Cellular mechanisms of cadmium-induced toxicity: a review. Int J Environ Health Res. 2014;24(4):378–99. doi: 10.1080/09603123.2013.835032 24117228

[23] Inaba T, Kobayashi E, Suwazono Y, Uetani M, Oishi M, Nakagawa H, et al. Estimation of cumulative cadmium intake causing Itai-Itai disease. Toxicology Letters. 2005;159:192–201. doi: 10.1016/j.toxlet.2005.05.011 16006079

[24] Organization WH. IPCS(Internation Programme on Chemical Safety) Environmental Health Criteria 135: Cadmium. Geneva, Switzerland.1992.

[25] 1999 AfTSaDRA. Toxicological profile for Cadmium Atlanta, GA: US Dept. of Health and Human Services, Public Health Services; 2012 [https://wwwn.cdc.gov/TSP/ToxProfiles/ToxProfiles.aspx?id=48&tid=15].

[26] Cadmium in Drinking Water: World Health Organization; 2011 [https://www.who.int/water_sanitation_health/dwq/chemicals/cadmium.pdf].

[27] Populations. NRCUCoMLiC. Measuring Lead Exposure in Infants, Children, and Other Sensitive Populations. Washington DC: National Academies Press (US); 1993 [https://www.ncbi.nlm.nih.gov/books/NBK236458/]. 25144057

[28] Slavin W. Accuracy in Furnace Atomic Absorption Spectroscopy. J Res Natl Bur Stand. 1988;93(3):445–6.

[29] Jorhem L. Determination of metals in foods by atomic absorption spectrometry after dry ashing: NMKL Collaborative Study. Journal of AOAC Int. 2000;83(5):1204–11. 11048861

[30] Ajteny Z, Bencs L, Haraszi R, Sziget J, Szoboszlai N. Study on the simultaneous determination of some essential and toxic trace elements in honey by multi-element graphite furnace atomic absorption. Talanta. 2007;71(683–690). 10.1016/j.talanta.2006.05.023 19071360

[31] Ruella de Oliveira S, Neto JAG. Evaluation of Bi as internal standard to minimize matrix effects on the direct determination of Pb in vinegar by graphite furnace atomic absorption spectrometry using Ru permanent modifier with co-injection of Pd/Mg(NO3)2. Spectrochimica Acta Part B. 2007;2007(1046–1050).

[32] Harvey D. Quality Assessment. Online: Northeastern University; 2019 [cited 2022. Available from: https://chem.libretexts.org/Courses/Northeastern_University/15%3A_Quality_Assurance/15.3%3A_Quality_Assessment.

[33] Acar O. Determination of cadmium an dlead in biological samples by Zeeman ETAAS using various chemical modifiers. Talanta. 2001;55:613–22. doi: 10.1016/s0039-9140(01)00468-4 18968407

[34] Maranhao TdA, Borges DLG, da Veiga MAMS, Curtius AJ. Cloud point extraction for the determination of cadmium and lead in biological samples by graphite furnace atomic absorption spectrometry. Spectrochemica Acta Part B. 2005;60(5):667–72.

[35] Erie JC, Butz JA, Good JA, Erie EA, Burritt MF, Cameron JD. Heavy metal concentrations in human eyes. Am J Ophthalmol. 2005;139(5):888–93. doi: 10.1016/j.ajo.2004.12.007 15860295

[36] Ortner HM, Bulska E, Rohr U, Schlemmer G, Weinbruch S, Welz B. Modifiers and coating in graphite furnace atomic absorption spectrometry-mechanisms of action (a tutorial review). Spectrochimica Acta Part B [Internet]. 2002; 57:[1835–53 pp.].

[37] Szkoda J, Zmudzki J. Determination of lead and cadmium in biological material by graphite furnace atomic absorption spectrometry method. Bulletin—Veterinary Institute in Pulawy. 2005;49:89–92.

[38] Blake C, Bourqui B. Determination of Lead and Cadmium in Food Products by Graphite Furnace Atomic Absorption Spectroscopy. Atomic Spectroscopy. 1998;19(6):207–13.

[39] Kingston HM, Jassie LB. Introduction to microwave sample preparation: Theory and practice.: American Chemical Society; 1988.

[40] Costa LM, Santos D, Hatje V, Nobrega JA, Korn MG. Focused microwave assisted acid digestion: Evaluation of losses of volatile elements in marine invertebrate samples. J of Food Composition and Analysis. 2009;22(3):238–41.

[41] Schmeling M, Gaynes B, Tidow-Kebritchi S. Heavy metal analysis in lens and aqueous humor of cataract patients by total-reflection X-ray fluorescence spectrometry. Powder Diff. 2014;29(155–158).

